# F-p Hybridization-Induced Ferromagnetism for Ultrathin Two-Dimensional Ferromagnetic Half-Metal (EuN) Monolayer: A First-Principles Study

**DOI:** 10.3390/molecules30102100

**Published:** 2025-05-09

**Authors:** Wenxue Sun, Yan Hu, Yuling Song, Yuhong Huang, Shuyao Cao

**Affiliations:** 1 School of Electronic Information, Huzhou College, Huzhou 313000, China; sunwenxue@zjhzu.edu.cn; 2 Huzhou Key Laboratory for Urban Multidimensional Perception and Intelligent Computing, Huzhou College, Huzhou 313000, China; 3 College of Physics and Electronic Engineering, Nanyang Normal University, Nanyang 473061, China; yuling985@163.com; 4 School of Physics & Information Technology, Shaanxi Normal University, Xi’an 710119, China; 5 School of Physics and Electronic Information, Yan’an University, Yan’an 716000, China

**Keywords:** ferromagnetism, ultrathin materials, rare-earth elements, curie temperature, magnetic anisotropy

## Abstract

By performing first-principles calculations, we predicted a kind of novel ultrathin two-dimensional (2D) ferromagnet, single-atomic-layer EuN. EuN monolayer is a ferromagnetic half-metal with a large band gap of 1.69 eV; Eu ions in EuN are in the highest spin state and have large magnetic moments of 6 μB, much larger compared with the non-rare-earth (RE) metal ions. The magneto-crystalline anisotropy energy (MCE) of EuN monolayer is −3.72 meV per Eu ion, which is much higher than that of CrI_3_ monolayer (0.685 meV per Cr ion); the magnetic dipolar energy (MDE) enhances magnetic anisotropy for EuN monolayer; large magnetic anisotropy energy (MAE) is beneficial to stabilizing the long-range ferromagnetic ordering. More importantly, different from many RE metal monolayers, hybridization between Eu-f and N-p orbitals induces ferromagnetism for EuN monolayer; the Curie temperature of EuN monolayer is above the liquid-nitrogen temperature (100 K). Additionally, the Curie temperature of EuN monolayer increases with increasing biaxial strain due to increased f-p hybridization.

## 1. Introduction

Along with the continuous miniaturization of electronic and spintronic devices, two-dimensional (2D) ferromagnetic (FM) materials have attracted intensive attention both theoretically and experimentally [1,2,3,4,5,6,7,8,9,10,11,12,13]. The first two 2D ferromagnets with thicknesses reaching the monolayer limit, CrI_3_ monolayer [14] and bilayer Cr_2_Ge_2_Te_6_ [15], were obtained in 2017. Subsequently, other monolayer-thick ferromagnets, Fe_3_GeTe_2_ and LnX_2_ (Ln = Eu, Gd; X = Si, Ge), were also synthesized [1,13,16]. Unfortunately, the Curie temperature for many synthesized 2D FM materials is lower [4,5,14,15], which restricts their practical applications. Many efforts have been devoted to design and achieve novel 2D ferromagnets with high Curie temperatures and large magnetic moments, which represent larger capacity, higher efficiency, and higher sensitivity [17,18,19,20,21].

Several methods have been adopted to increase the Curie temperature of 2D ferromagnets [11,12,18,19,20]. Reducing the band gap and modulating the virtual hopping gap for ferromagnets lead to stronger FM coupling dominated by super-exchange interaction between equivalent magnetic ions [18]. Additionally, inducing extra interlayer FM interaction [11] and tuning bond length [12] both were used to increase the Curie temperature.

On the other hand, the local magnetic moment of magnetic ions in transition-metal (TM) FM materials should be less than 5 μB due to the restriction of the Goodenough–Kanamori–Anderson (GKA) rule [22]. The predicted local magnetic moments in the TM FM materials are 0.45~4.5 μB. Both FM Mn_2_NF_2_ and Mn_2_N(OH)_2_ MXenes [23] have the largest magnetic moment of 4.5 μB per Mn ion. Via H adsorption [24] or inducing Mn atom vacancies [25], MnBr_2_ monolayer converts from AFM to FM; each Mn ion breaks the d^5^ configuration and, thus, has a large magnetic moment of 4 μB. Since the f-shell can contain seven valence electrons, rare-earth (RE) metal monolayers can have larger magnetic moments, for example, the magnetic moment of each Gd ion for Gd_2_B_2_ [26] monolayer can reach up to 7.30 μB.

Since two RE metal Eu-based 2D ferromagnets, EuSi_2_ [16] and EuGe_2_ [13], have been synthesized, the variety of RE metal monolayers have gradually become enriched both experimentally [13] and theoretically [17,26,27,28,29,30,31]; their novel physical and chemical properties such as ferro-elasticity [27], topological properties [28], and valley polarization [29,30,31], are also being studied. Among them, many FM monolayers have large magnetic moments (7.3~8 μB/Gd ion) [17,26,27,29,30,31] and high Curie temperature (241~550 K) [17,26,29]; previous studies have indicated p-d hybridization between Gd and sp-element ions, playing an important role in inducing ferromagnetism since f electrons are highly localized and far away from the Fermi level [17,27,31]. Moreover, heavier RE metal ions usually have stronger spin-orbit coupling (SOC) and larger magneto-crystalline anisotropy energy, which are beneficial for stabilizing long-range magnetic order [17,26,27,28,29,30]. However, whether any hybridization mechanism can induce ferromagnetism for RE metal monolayers is still unknown; moreover, more RE metal monolayers with large magnetic moments and high Curie temperature need to be explored.

Additionally, EuN, cubic non-layered rare-metal thin films were synthesized [32,33], implying the possibility of another Eu-based 2D ferromagnetic monolayer. Previous studies have theoretically investigated electronic and magnetic properties for bulk EuN in the NaCl structure using the local spin density approximation (LDA) [34], LDA+U [33,34,35,36], dynamic mean field theory (DMFT) [33], quasiparticle self-consistent GW (QSGW) calculations [33], and generalized gradient approximation (GGA)+U methods [37]. All these methods indicate that bulk EuN is FM and Eu ions are in the highest spin states, but there have not been any investigations, both theoretically and experimentally, for EuN monolayer. Different from other studied RE metal ferromagnetic monolayers [17,27,28,29,30,31], EuN monolayer only has a one-atomic-thick layer; Eu ions are likely to yield larger magnetic moments due to more f electrons. In this paper, we comprehensively studied EuN monolayer via adopting a first-principles method based on the density functional theory (DFT). Our calculations show that EuN monolayer is a ferromagnetic half-metal with a large magnetic anisotropy energy (MAE) of −4.24 meV per formula unit and a large magnetic moment of 6 μB per Eu ion. The Curie temperature for EuN monolayer is higher than the liquid-nitrogen temperature (100 K) under 1~5% biaxial strain; its Curie temperature can increase with increasing tensile strain. Moreover, different from other RE metal monolayers whose ferromagnetism originated from d-p hybridization [17,27], f-p hybridization is the key to induce ferromagnetism for EuN monolayer.

## 2. Results and Discussion

### 2.1. Ground State, Stability, Electronic and Magnetic Properties of EuN Monolayer

**Figure 1:**
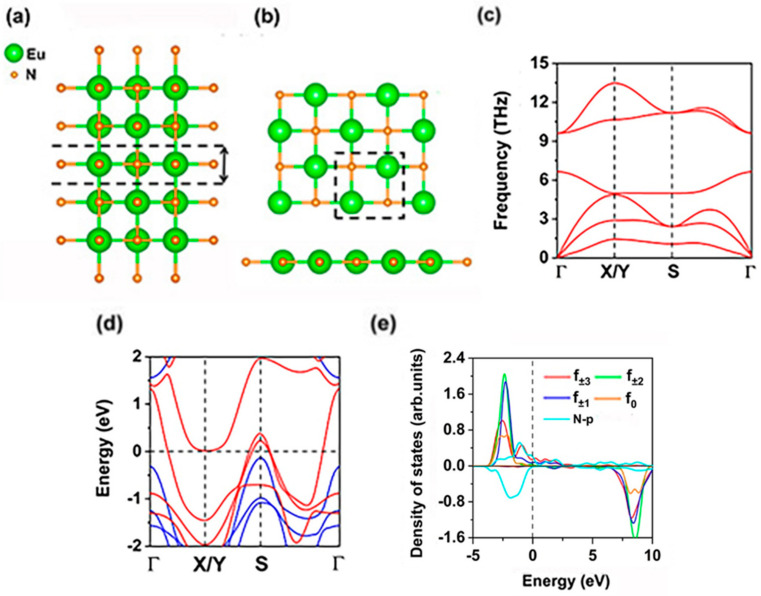
(**a**) Bulk EuN and (**b**) the top and side views of EuN monolayer. (**c**) Phonon spectra, (**d**) electronic band structure, and (**e**) projected densities of states (DOSs) of Eu-4f orbitals and N-2p orbitals of EuN monolayer. The primitive cell circulates in dash lines. The Fermi level is set as 0 eV.

**Figure 2:**
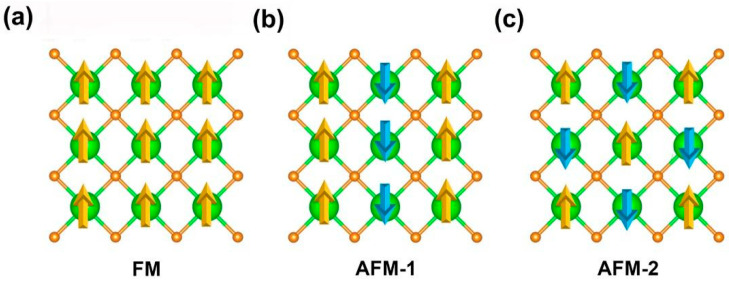
(**a**) Ferromagnetic (FM) and two antiferromagnetic (**b**) AFM-1 and (**c**) AFM-2 configurations of EuN monolayer.

In the GGA+U method, for NaCl, CsCl, and Zinc-Blende structures, the most stable structure for cubic bulk EuN at the equilibrium lattice is the NaCl structure [37]; high crystalline quality epitaxial growth of EuN thin films with NaCl structure was realized [33]. EuN monolayer may be obtained based on thin films with this structure, as shown in Figure 1a, like TiN and VN thin films. We also hope EuN monolayer can be prepared via the efficient chemical vapor deposition (CVD) [38,39,40] or sputter process methods [41,42,43]. As shown in Figure 1b, EuN monolayer is planar, like graphene and h-BN monolayers, and has the space group of *P*4/*MMM* (No. 123), whose lattice along the x and y directions are isotropic. Firstly, ferromagnetic (FM) and two antiferromagnetic (AFM-1, AFM-2) configurations were simulated by the 2 × 2 × 1 supercell shown in Figure 2. The results show that the ground state of monolayer EuN is FM, which is more stable than the AFM-1 and AFM-2 states by 17.0 and 129.6 meV per formula unit, respectively (Table S1). In the following, we only discuss the properties of EuN monolayer in the FM ground state. The bulk EuN is also FM based on LDA, LDA+U, GGA+U, DMFT, and QSGW methods [33,34,35,36,37]. Its unit cell is square with lattice constants of a = b = 3.38 Å; Eu and N ions are arranged interlaced with four nearest neighboring (NN) N and Eu ions, respectively, and the distance between the NN Eu and N ions is 2.38 Å.

Secondly, we examined the stability of the EuN monolayer. Figure 1c displays the phonon spectra, showing no imaginary frequencies and dynamical stability. There are only two independent elastic constants for EuN monolayer due to the lattice symmetry, namely, *C*_11_ and *C*_12_. The *C*_11_ and *C*_12_ are 32.36 J/m^2^ and 5.10 J/m^2^, respectively; the elastic constant $C_{22}$ is equal to $C_{11}$, and $C_{66}=\frac{1}{2}(C_{11}-C_{12})$; thus, $C_{22}$ and $C_{66}$ are 32.36 J/m^2^ and 13.63 J/m^2^, respectively, satisfying the Born–Huang criteria [44] of $C_{11}>0$, $C_{11}C_{22}-C_{12}^{2}>0$, and $C_{66}>0$, indicating that EuN monolayer is mechanically stable. The Young’s modulus is 26.62 J/m^2^, which is comparable to that of the synthesized CrI_3_ monolayer (23.85 J/m^2^) [45], indicating EuN monolayer can maintain its free-standing structure without any curling. The gravity-induced out-of-plane deformation h can be evaluated via the Young’s modulus as follows [46]:

$$\frac{h}{L}\approx {\left(\frac{\rho gL}{Y}\right)}^{1/3}$$

For EuN monolayer where the mass density ρ is $4.70\times 10^{-6}\;{\mathrm{kg}/m}^{2}$, L is the size of the monolayer, and the acceleration of gravity g is ${9.8\;m/s}^{2}$. Taking L≈100 μm, we obtain $\frac{h}{L}\approx 6.97\times 10^{-4}$; this magnitude is the same as that of synthesized monolayer graphene [46]; thus, the predicted induced deformation is negligible, although EuN monolayer is ultrathin.

The formation energy was further calculated as follows:

*E*_*form*_ = *E*_*EuN*_ − *E*_*Eu*_ − *E*_*N*_

where *E_EuN_* represents the energy of EuN monolayer, *E_Eu_* and *E_N_* are the atomic energies of BCC (body center cubic) bulk Eu and N_2_ gas, respectively. The calculated *E_form_* is −1.185 eV. The negative value of *E_form_* means that the formation of EuN monolayer is exothermic and energetically favorable.

Further, the spin-resolved electronic band structure shown in Figure 1d indicates that EuN monolayer is a kind of novel half-metal. The majority spin-channel is metallic while the minority spin-channel exhibits semiconducting features with a large energy gap of 1.69 eV. The electronic states at the valence band maximum (VBM) and conduction band minimum (CBM) are contributed by the N and Eu ions, the VBM and CBM are −0.13 eV and 1.56 eV, respectively. For bulk EuN with NaCl structure, the half-metallicity was verified based on LDA, LDA+U, and GGA+U methods [33,34,35,36,37], but for DMFT and QSGW methods, bulk EuN is a semimetal or semiconductor [33]. Additionally, the orbital-resolved densities of states (DOSs) indicate that the Eu-4f orbitals are decomposed due to the *D_*4h point group of the square planar lattice. According to the magnetic quantum number, the seven Eu-4f orbitals split into *f* ± 3 (*f*(*x*(*x*^2^ − 3*y*^2^)), *f*(*y*(3*x*^2^ − *y*^2^))), *f* ± 2 (*f*(*xz*^2^), *f*(*yz*^2^)), *f* ± 1 (*f*(*xyz*), *f*(*z*(*x*^2^ − *y*^2^))), and *f*0 (*f*(*z*^3^)) groups. Plus, both majority and minority spin-channels of the three N-2p orbitals are fully occupied. The Bader charge analysis suggests that each Eu ion donates 2.55 electrons to the neighboring N ions. Our results indicate that each N atom is in the 2p^6^ configuration by gaining three valence electrons from each Eu atom; each Eu ion has six 4f valence electrons and is located in the high spin state. Occupied f electrons all fill in the spin-up channel, leaving the seven spin-down 4f orbitals unoccupied, as shown in Figure 1e. Correspondingly, the spin-splitting and magnetic moment of Eu ions are large (6 μB), there are a lack of d electrons for each Eu ion, and the valence electrons configurations for both Eu and N ions are 4f^6^ and 2s^2^2p^6^, respectively. Previous experimentation has indicated that Eu^3+^ ions dominate for EuN thin films, which is consistent with our results, but several Eu^2+^ ions can be generated because of defects [33]; calculation results based on LDA+U, GGA+U, QSGW, and DMFT methods have indicated both Eu^2+^ and Eu^3+^ ions have large spin magnetic moments of 6 μB and 7 μB, respectively [33,34,35,36,37].

The orbital-projected band structure (Figure S1) also indicates p-d or f-d hybridizations near the Fermi level is negligible; the total magnetic moment of EuN monolayer is 6.12 μB per unit, mainly contributed by the Eu ion, consistent with the spin-charge distributions (SCD) shown in Figure S2.

### 2.2. Curie Temperature and Magnetic Anisotropy Energy of EuN Monolayer

We considered the magnetic coupling between the NN and second NN (2NN) Eu ions. The spin-Hamiltonian including the NN and 2NN exchange interaction is described as follows [10]:

$$H=-\sum_{i,j}J_{1}M_{i}M_{j}-\sum_{i,j}J_{2}M_{i}M_{j}$$

where *J*_1_ and *J*_2_ are the NN and 2NN magnetic exchange parameters, respectively; *i* and *j* stand for the NN and 2NN pair of Eu ions, respectively; and *M_i_* and *M_j_* are the corresponding net magnetic moments of the NN and 2NN pair of Eu ions, respectively. The magnetic coupling parameters are calculated via the energy difference between the FM and two AFM states in the 2 × 2 × 1 supercell as follows:

$$J_{1}=\frac{E_{AFM2}-E_{FM}}{16M^{2}}$$

$$J_{1}+2J_{2}=\frac{E_{AFM1}-E_{FM}}{8M^{2}}$$

The calculated *J*_1_ and *J*_2_ are 0.45 meV and −0.11 meV, respectively. The positive/negative value of *J* indicates the preference for FM/AFM coupling.

**Figure 3:**
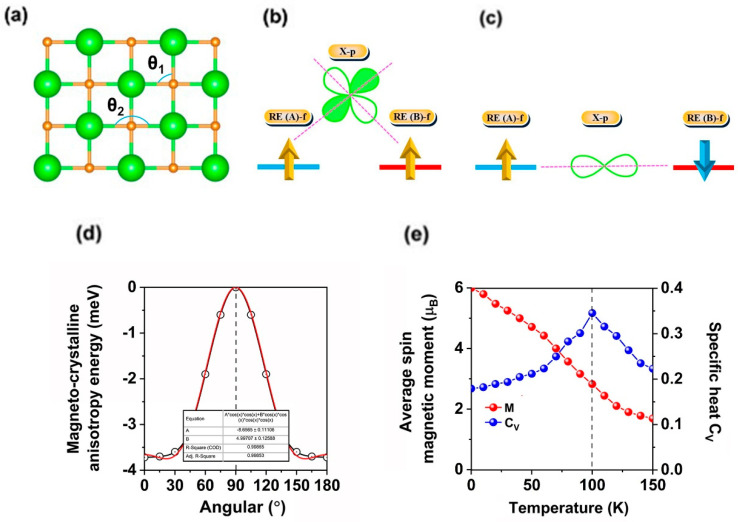
(**a**) The magnetic coupling between the nearest neighboring (NN) and second nearest neighboring (2NN) magnetic ions. *θ*_1_ and *θ*_2_ represent the Eu-N-Eu angles on the super-exchange paths of NN and 2NN coupling, respectively. Super-exchange interaction when the Eu-N-Eu angles are (**b**) 90° and (**c**) 180°. (**d**) The variation of magneto-crystalline anisotropy energy (MCE) of EuN monolayer with respect to azimuthal angle. The red solid line is the fitted line, the inset-table lists the standard errors for the fitting slope. (**e**) Variation of on-site magnetic moments of Eu ions and the specific heat *C_v_* of EuN monolayer with temperature.

Since the spin states for each Eu ion are nearly half-filled, the direct magnetic coupling between the neighboring Eu ions is FM according to the Hund’s rule [47], and the indirect magnetic coupling between the neighboring Eu ions mediated via N ions is super-exchange interaction since all Eu ions are equivalent. According to the Goodenough–Kanamori–Anderson (GKA) rule [22], two Eu ions ferromagnetically couple with each other via different p orbitals of the mediated N ion when the Eu-N-Eu angle is close to 90° (Figure 3b). In this case, the super-exchange interaction is FM, but when the Eu-N-Eu angle is close to 180°, two Eu ions couple with the same p orbital of the mediated N ion; as a result, super-exchange is AFM (Figure 3c) [22]. As for NN Eu pairs, both super-exchange and direct exchange interactions are FM since *θ*_1_ is 90°. As for the second NN Eu ions, there are two kinds of interaction: direct-exchange, and the AFM super-exchange path along the x or y directions with the 180° Eu-N-Eu angle (*θ*_2_) (Figure 3a). Since Eu-Eu bonds along the direct-exchange paths between the 2NN ions are much longer than those of the NN Eu ions, FM direct interactions between 2NN Eu pairs are also correspondingly weaker. AFM super-exchange interaction competes over the FM direct-exchange; thus, 2NN Eu ions are AFM coupling, since AFM interactions between 2NN Eu ions are smaller than FM interactions between NN Eu ions; the ground state for EuN monolayer is still FM. Moreover, different from monolayer GdI_2_ [17], f electrons for Eu ions are close to the Fermi level; thus, f-p-f super-exchange interactions between Eu and N ions can happen without using d electrons as a medium.

Magneto-crystalline anisotropy energy (MCE) is the other important parameter for the realization and maintenance of long-range FM ordering at high temperature. The MCE of EuN monolayer was calculated by comparing the total energies of the monolayer with the magnetization axis along the x/y direction and along the z direction by considering the spin-orbit coupling (SOC). The total energies with the magnetization axis along the x and y directions are equal due to the lattice symmetry. The easy magnetization axes are along the x/y directions with an MCE of −3.72 meV per formula unit; this value is much larger than that of the synthesized CrI_3_ monolayer (0.685 meV) [45] and many RE metal FM monolayers, including GdI_2_ (553 µeV) [17], GdS_2_, GdSe_2_, GdSSe (134~1078 µeV) [27], and 2H-GdIBr (−0.42 meV) [29], ensuring long-range ferromagnetic ordering. The MCE can also be described by series expansions in terms of the azimuthal angle as follows [48]:

*MCE*(*θ*) = *A*cos^2^ (*θ*) + *B*cos^4^ (*θ*)

where *A* and *B* are the anisotropy constants, and *θ* is the azimuthal angle between the magnetization axis and x/y direction. The fitting results are shown in Figure 3d, which shows that cos^2^ (*θ*) is smaller than −*A*/*B*; thus, the magnetization is along the in-plane directions.

Moreover, considering the large magnetic moment of Eu ions, we also calculated the magnetic dipolar energy (MDE) for EuN monolayer. For an FM system, the MDE in atomic Rydberg units is given by the following [49,50]:

$$E_{d}=\sum_{ij}\frac{2m_{i}m_{j}}{c^{2}}M_{ij}$$

where the speed of light c=274.072, i/j are the atomic position vectors in the unit cell, and $m_{i}/m_{j}$ is the atomic magnetic moment (in units of μB) on site i/j. The magnetic dipolar Madelung constant $M_{ij}$ is calculated via the following:

$$M_{ij}=\sum_{R}\frac{1}{{\left|R+i+j\right|}^{3}}\left\{1-3\left.\frac{{\left[(R+i+j)\cdot \overset{\wedge }{m_{i}}\right]}^{2}}{{\left|R+i+j\right|}^{2}}\right\}\right.$$

where R are the lattice vectors. Using the calculated magnetic moments, the (shape anisotropy energy) is obtained as the difference in $E_{d}$ between the in-plane and out-of-plane magnetizations; the calculated $\Delta E_{d}$ is −0.52 meV for each Eu ion, which indicates that magneto-static dipole–dipole interaction strengthens in-plane magnetic anisotropy. For many magnets, total magnetic anisotropy energy (MAE) is the sum of MCE and MDE; the value of MAE is −4.24 meV per formula unit for EuN monolayer, larger than that of monolayers FeI_3_ (−2.16 meV) [12], V_2_X_3_ (X = O, S, Se) (200~260 µeV) [51], EuSn_2_X_2_ (X = P, As) (−295, −354 µeV) [52], and zigzag chains of Fe, Cr, and Ni (1.51~2.94 meV) [53].

In the present study, *T_C_* of EuN monolayer was investigated by performing the Monte Carlo (MC) simulations based on the Heisenberg model. The 100 × 100 × 1 supercell containing 20,000 magnetic moment vectors was adopted to perform the MC simulations, which last for 10^5^ steps at each temperature. Each magnetic moment vector is rotated randomly. We adopted the same method to estimate *T_C_* of CrI_3_ monolayer, which is 42 K [11,12], agreeing well with the experimental measured value [14] and previous calculation results [17,18,19,20,26]. Figure 3e shows the evolution of specific heat defined as *C_V_* = (<E^2^>-<E>^2^)/K_B_T^2^ with temperature, from which we obtained the *T_C_* of 100 K for EuN monolayer by locating the peak position of *C_v_*, obtaining the curve in Figure 3e without any leaps, indicating that the simulated parameters are accurate. The evolution of the atomic magnetic moments of Eu ions with temperature yields the same *T_C_*, which is higher than that of the liquid-nitrogen temperature (77 K), but still under room temperature. For many studied RE metal ferromagnetic monolayers [17,26,27,28,29,30,31,52], this value is higher than that of EuSn_2_X_2_ (X = P, As) (17, 27 K), GdS_2_, GdSe_2_, and GdSSe (43~68 K) monolayers [27,52]. The *T_C_* was also estimated using a mean-field approximation (MFA) [54,55], and the obtained *T_C_* was 249 K; similar to other 2D materials [56], the MFT overestimates *T_C_*.

According to the Fermi–Dirac distribution [57], the thermally induced occupation probability between the valence band and the Fermi level is on the order of 10^−7^ at the Curie point for EuN monolayer.

### 2.3. Tunable Electronic and Magnetic Properties Under Biaxial Strain

**Figure 4:**
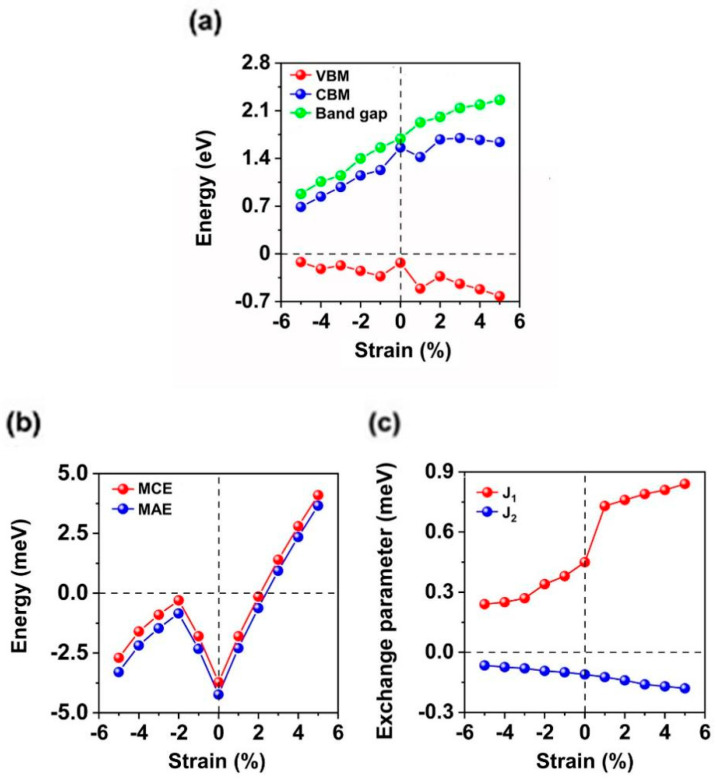
(**a**) The valence band maximum (VBM), conduction band minimum (CBM), and band gap for the spin-down channel. (**b**) Magneto-crystalline anisotropy energy (MCE), magnetic anisotropy energy (MAE) of Eu ions, and (**c**) nearest, next-nearest magnetic-exchange parameters $J_{1}$, $J_{2}$ of EuN monolayer under biaxial strain.

We tried to increase the *T_C_* of EuN monolayer by applying biaxial strain, which can be realized experimentally via bending flexible substrates [58,59,60,61], elongating elastic substrate [62,63], exploiting the thermal expansion mismatch [64], and so on. Under biaxial strain from −5% to 5%, the EuN monolayer maintains as a half-metal, and the band gap of the semiconducting spin-channel increases from 0.88 to 2.27 eV (Figure 4a); the positions of VBM range from −0.62 to −0.12 eV while the positions of CBM range from 0.69 to 1.71 eV, respectively. The easy magnetization axes change to be along the z direction under 3~5% biaxial strain, with the MCE reaching a maximum value of 4.10 meV under 5% strain (Figure 4b). The value of MAE is smaller than MCE under 3~5% biaxial strain due to in-plane MDE and reaches a maximum value of 3.66 meV at 5% strain.

**Figure 5:**
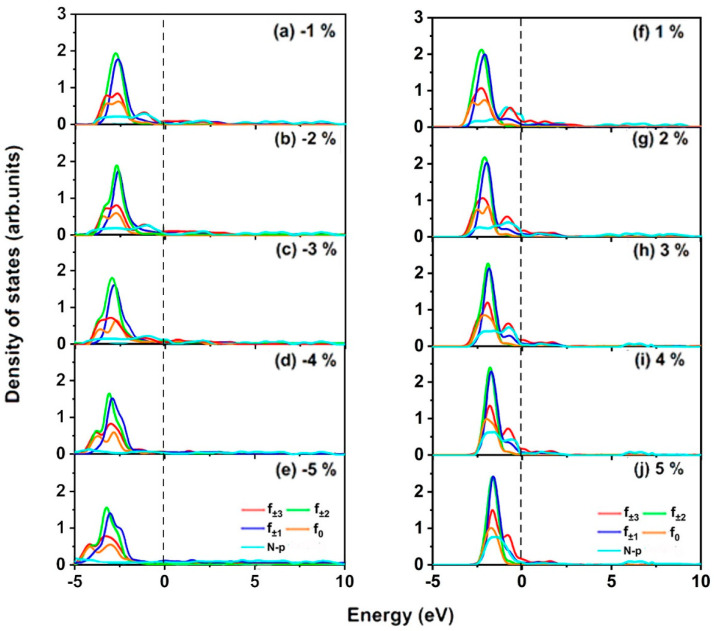
(**a**–**j**) Projected densities of states (PDOSs) in the spin-up channel of Eu-f and N-p orbitals under biaxial strain from −5 to ~5% for EuN monolayer; the Fermi level is set as 0 eV.

Moreover, *J*_1_ increases with increasing biaxial strain, while *J*_2_ is still negative and decreases with increasing strain (Figure 4c). The variations in magnetic exchange parameters *J*_1_ and *J*_2_ under tensile biaxial strain can be explained as follows:

Firstly, with increased strain, all the occupied spin-up channels of both f- and p-orbitals become more localized. Meanwhile, these orbitals all move close to the Fermi level, leading more electrons to gather around the Fermi level (Figure 5f–j). As shown in Figure 5f–j, more occupied localizations around the Fermi level benefit f-p hybridization between Eu and N ions, especially for the hybridization between N-p and Eu-*f* ± 2 (*f*(*xz*^2^), *f*(*yz*^2^)), *f* ± 1 (*f*(*xyz*), and *f*(*z*(*x*^2^ − *y*^2^)) orbitals. Hence, the FM super-exchange interaction for NN Eu ions under tensile strain enhances. On the contrary, the spin-up channels of f-orbitals become increasingly delocalized, and all of them move away from the Fermi level under compressive strain from −1% to −5% (Figure 5a–e); hybridization between N-p and Eu-*f* ± 2 (*f*(*xz*^2^), *f*(*yz*^2^)), *f* ± 1 (*f*(*xyz*), and *f*(*z*(*x*^2^ − *y*^2^)) orbitals decreases more rapidly. Consequently, both f-p hybridization and the FM super-exchange interaction are weaker between NN Eu ions with increased compressive strain.

**Figure 6:**
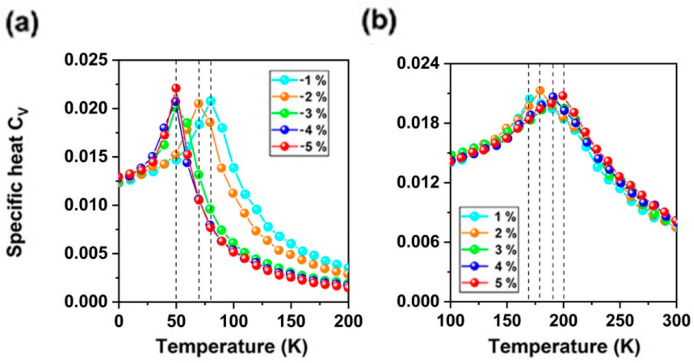
(**a**) The specific heat *Cv* of EuN monolayer under (**a**) −1%, −2%, −3%, −4%, −5% compressive strain, and (**b**) 1%, 2%, 3%, 4%, 5% tensile strain as function of temperature based on Heisenberg model.

Secondly, under biaxial strain from −5% to 5%, the 2NN Eu-Eu bonds lengthen. As we know, longer Eu-Eu bonds weaken direct FM interaction, thus *J*_2_ decreases due to the much weaker FM direct-exchange. On the other hand, with increasing biaxial strain, stronger Eu-N hybridization strengthens the AFM super-exchange interaction between 2NN Eu ions; thus, with increasing biaxial strain, both stronger AFM super-exchange interaction and weaker FM direct interaction result in decreased *J*_2_. Although longer NN Eu-Eu bonds can also weaken direct FM interaction with increased strain, enhanced FM super-exchange interaction dominates for NN Eu ions and *J*_1_, since *J*_2_ is still smaller than *J*_1_. The *T_C_* of EuN monolayer is increased with both *J*_1_ and increased biaxial strain (Figure 6). At 5% strain, the *T_C_* of EuN monolayer can reach up to 200 K, while under biaxial strain from −5% to −2%, it falls below the liquid-nitrogen temperature (Figure 6). At the Curie point [65], the thermally induced occupation probabilities between the valence band and Fermi level are on the orders of 10^−13^, 10^−23^, 10^−18^, 10^−19^, 10^−21^, 10^−16^, 10^−10^, 10^−12^, 10^−14^, and 10^−16^ under −5~5% biaxial strain for EuN monolayer.

### 2.4. Discussion About Experimental Fabrication of EuN Monolayer

Although EuN thin films have been obtained and bulk EuN has been theoretically investigated extensively [33,34,35,36,37], there are not any studies for EuN monolayer. Currently, the Eu metal can be prepared by the reduction of high-purity Eu_2_O_3_, and then placed into an Mo boat, which is placed in a Vycor tube with many Ti turnings; the reaction between Eu and N occurs when N_2_ is warmed through the system mentioned above. Finally, this system is sealed off and heated for 12–16 h at 1074 K; in this way, EuN sample with NaCl-type structure can be obtained [65]. High crystalline quality EuN thin films 75 nm thick can epitaxially grow on (100)-oriented yttria-stabilized zirconia (YSN) successfully, the growth of EuN is sensitive to the growth conditions, and the nitrogen source must be excited [33]. Measured results indicate that EuN thin films are not a layered material; thus, using micro-mechanical exfoliation methods to obtain EuN monolayer is challenging. By employing the molecular-beam epitaxial (MBE) method, controlled growth of epitaxial layers of different thickness can be realized, thus we hope EuN monolayer can also be synthesized using the MBE method for LnX_2_ (Ln = Gd, Eu; X = Si, Ge) monolayers [13] and for LnX_2_ (Ln = Gd, Eu; X = Si, Ge) monolayers. The surface of the X substrate comes into contact with a flow of Ln atoms; the growth process happens at higher temperature. Moreover, for CrN (001) thin films whose structure is similar to EuN monolayer [66], based on the physical vapor deposition (PVD) method, using a Cr disk as a target, CrN films were deposited onto the MgO-(001) substrates under irradiation with nitrogen radicals. This method also has potential for synthesizing EuN monolayer. Considering the reactivity of Eu ions, monolayer EuNX_2_ (X = F, O, H, etc.) may be generated; thus, the growth process should take place in the glovebox and the obtained EuN monolayer should be covered in an inert environment immediately.

## 3. Computational Methods

All the calculations in the present study were performed by adopting the spin-polarized density function theory (DFT) as implemented in the Vienna ab initio simulation package (VASP) [67]. Interactions between electrons and nuclei were described by the projector augmented wave (PAW) method [68,69], and the electronic exchange-correlation interactions were described by the Perdew–Burke–Ernzerhof (PBE) functional of the generalized gradient approximation (GGA) [70] method. Particularly, the more accurate Heyd–Scuseria–Ernzerhof (HSE06) method [71] with the screening parameter fixed at a value of 0.2 Å^−1^ was adopted to calculate the electronic band structure and density of states. The Brillouin zone integration was carried out by a sufficiently large 9 × 9 × 1 k-mesh based on the Monkhorst–Pack scheme [72]. A vacuum space of 20 Å was added along the direction perpendicular to the surface of the monolayer to avoid interactions between the adjacent layers. The cutoff energy for the plane-wave basis set was set as 500 eV. The phonon spectra were calculated using the Phonopy code [73], which is implemented within the VASP package. The convergence criterion for the total energy and force were set as 1 × 10^−8^ eV and 0.001 eV/Å, respectively.

## 4. Conclusions

In summary, we predicted a novel two-dimensional (2D) one-atomic-thick ferromagnetic half-metal: EuN monolayer, whose stability was predicted comprehensively. The total magnetic moment of EuN monolayer is 6.12 μB per formula unit, and its easy magnetization axis is along the in-plane directions with an MAE of −4.24 meV per Eu ion. The predicted Curie temperature of EuN monolayer is 100 K, higher than the liquid-nitrogen temperature; f-p hybridization between Eu and N ions results in ferromagnetism for EuN monolayer. Under biaxial strain from −5% to 5%, the half-metallicity can be preserved, and the Curie temperature for EuN monolayer increases with increasing biaxial strain due to stronger f-p hybridization. Additionally, the effects of Eu^2+^ ions, dangling bonds, substrates, and surface passivation need to further studied.

## Data Availability

The original contributions presented in this study are included in the article/Supplementary Material. Further inquiries can be directed to the corresponding author.

## Supplementary Materials

The following supporting information can be downloaded at: https://www.mdpi.com/article/10.3390/molecules30102100/s1.
