# Supplementary material for: F-p Hybridization-Induced Ferromagnetism for Ultrathin Two-Dimensional Ferromagnetic Half-Metal (EuN) Monolayer: A First-Principles Study
Source: Molecules. 2025 May 9;30(10):2100. doi: 10.3390/molecules30102100 (PMC12113815; doi:10.3390/molecules30102100)
Supplement: Supplementary file 1 [file molecules-30-02100-s001.zip › molecules-3530960-supplementary.pdf]

Table S1. The calculated energies of the ferromagnetic (FM) and two anti-ferromagnetic (AFM-1, AFM-2) configurations relative to the nonmagnetic (NM) configuration for EuN monolayer.

	NM/eV	FM/eV	AFM-1/eV	AFM-2/eV
EuN	0	-3.007	-2.990	-2.942

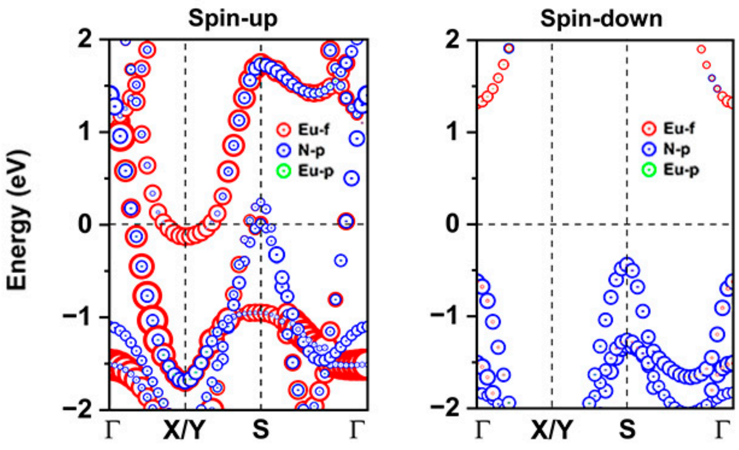

Figure S1. Orbitals-projected band structures for EuN monolayer.

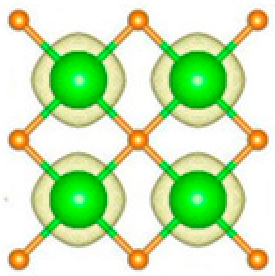

Figure S2. Spin-resolved charge density (SCD) of EuN monolayer with the isosurface value set as 0.03 eÅ^{-3}.
